# The effect of gemigliptin treatment on immune parameters including regulatory T cells in patients with type 2 diabetes and moderate to very severe chronic renal impairment

**DOI:** 10.1097/MD.0000000000036455

**Published:** 2023-12-08

**Authors:** Yanghyeon Kim, Nagyeom Lee, Sujung Heo, Ye Na Kim, Ho Sik Shin, Yeonsoon Jung, Hark Rim

**Affiliations:** a Renal Division, Department of Internal Medicine, Gospel Hospital, Kosin University College of Medicine, Busan, South Korea; b Transplantation Research Institute, Kosin University College of Medicine, Busan, South Korea.

**Keywords:** CKD, DM, gemigliptin, kindey transplantation, regulatory T cells

## Abstract

It is crucial to understand the impact of DPP-4 inhibitors on the immune system, particularly T cell differentiation, maturation, and proliferation, in patients with type 2 diabetes and CKD. This prospective observational study aimed to investigate the distribution of immune cells (particularly regulatory T cells), following the administration of gemigliptin, a DPP-4 inhibitor, in patients with type 2 diabetes mellitus and chronic kidney disease. We enrolled 28 patients with type 2 diabetes, aged 20 to 69, who had been taking a daily dose of 50mg gemigliptin for <3 months and had chronic kidney disease stages 3, 4, or 5, including that undergoing dialysis. T regulatory cells were defined as CD4 + CD25 high CD127 low/- FoxP3 + phenotype, and flow cytometry was used to examine the distribution of T regulatory cells. In the patient group, blood samples were collected at baseline, as well as at 3 and 6 months after initiating medication. Of the 28 patients, 17 (60.7%) were male and the mean age was 61.82 ± 8.03 years. Serum Cr ≥ 1.5 mg/dL was 16 (57%), and Cr < 1.5 mg/dL was 12 (43%). The number of CD4(+)/CD25(+) cells did not significantly increase or decrease in baseline, 3 months, and 6 months time changes, and the number of CD127(-/FoxP3(+) cells did not change significantly. Treatment with gemigliptin for 3 and 6 months did not significantly alter the number, percentage, or ratio of circulating Treg cells in patients with type 2 diabetes and CKD. Therefore, the administration of gemigliptin may help maintain regulatory T cells or have no significant impact.

## 1. Introduction

Dipeptidyl peptidase (DPP)-4, also known as CD26, is a multifunctional cell-surface glycoprotein expressed in various cells and tissues. It serves as a peptidase, protein binder, and T cell co-stimulator. DPP-4 plays a significant role, particularly in association with T cells.^[1]^

DPP-4 is a multifaceted glycoprotein involved in various T cell signaling pathways and serves as a marker of T cell activation. Initially identified as a CD4 + T cell activation antigen, CD26/DPP-4 is prominently expressed on activated T cells, especially proinflammatory T-helper (Th) type 1 and 17 cells, while anti-inflammatory subsets like Th2 and regulatory T cells (Treg cells) exhibit lower CD26/DPP-4 expression. A soluble form of CD26 (sCD26) found in serum displays DPP-4 enzymatic activity, removing X-Pro and X-Ala dipeptides from substrates and inducing T cell proliferation in response to mitogens or recall antigens. It is plausible that CD26/DPP-4 plays a role in regulating T cell differentiation, maturation, proliferation, and T cell-dependent immune functions. Additionally, CD26/DPP-4 rapidly cleaves N-terminal dipeptides of incretin hormones (GLP-1 and GIP), degrading their insulinotropic activity within minutes.^[1,2]^

DPP-4 inhibitors are a class of antidiabetic drugs that enhance the action of endogenous incretin hormones (GLP-1 and GIP). Their clinical use is rapidly increasing worldwide. CD26/DPP-4 regulates various chemokines involved in immune system modulation. As a result, DPP-4 inhibitors may impact the immune system by reducing the pathological effects of Th1 and Th17 cells, upregulating Th2 cells, and promoting the function of T regulatory cells.^[1–4]^

Gemigliptin is a well-tolerated, long-acting inhibitor of DPP-4, which is reversible, potent, selective, and competitive. Its use is not associated with an increased risk of hypoglycemia or changes in body weight.^[5–7]^

Additionally, there is a report indicating that in patients undergoing dialysis due to end-stage renal failure, a higher composition ratio of regulatory T cells is associated with a lower rejection rate following kidney transplantation.^[8,9]^

Regulatory T cells (Tregs) are crucial for maintaining immune homeostasis and preventing autoimmune diseases, including those affecting the kidneys. They likely also play a role in reducing kidney transplant rejection and potentially promoting transplant tolerance. Among various Treg subsets, the most potent and well-defined Tregs are those expressing Foxp3, which originate from the thymus or are generated in peripheral tissues.^[8–10]^

Patients in CKD stages 3, 4, and 5 often face the likelihood of progressing to terminal renal failure. Kidney transplants offer hope for an improved quality of life for those who require dialysis.

Kidney transplantation is a standard life-saving therapy for end-stage kidney disease. However, a significant number of kidney grafts are lost within 10 years due to chronic rejection, immunosuppressive drug-related nephrotoxicity, and cumulative side effects, including increased risks of heart disease, infection, cancer, and diabetes. Achieving kidney transplant tolerance is a major clinical goal. Observational studies suggest that Tregs, particularly CXCR3^+^ and HLA-DRhigh^+^ CD45RA^−^ Tregs in peripheral blood that produce high levels of interferon-g, are associated with improved renal allograft survival and function.^[8,9]^

Enhancing our understanding of factors that promote or inhibit Treg stability in both homeostatic and inflammatory contexts may lead to alternative strategies for achieving long-term graft survival.^[8,9]^

It is crucial to understand the impact of DPP-4 inhibitors on the immune system, particularly T cell differentiation, maturation, and proliferation, in patients with type 2 diabetes and CKD.

While studies have examined immune cell changes after 3 months of using a DPP-4 inhibitor (sitagliptin) in type 2 diabetes patients,^[1,4]^ there is currently no research on the impact of a DPP-4 inhibitor on the immune system at 3 and 6 months in type 2 diabetes patients with chronic kidney disease.

We conducted a prospective observational study to examine immune cell distribution, with a focus on regulatory T cells, following the administration of gemigliptin, a DPP-4 inhibitor, in patients with type 2 diabetes mellitus (T2DM) and chronic kidney disease. The study aimed to assess changes in immune cell composition among type 2 diabetic patients with CKD who had been taking gemigliptin (50mg daily) for less than 3 months.

## 2. Methods

We studied 28 patients (17 women and 11 men) in Kosin university gospel hospital. This study is a prospective observational study with no control group established.

The patient group is T2DM with a fasting blood glucose level of 126 mg/dL or higher, or a blood glucose level of 200 mg/dL or higher 2 hours after a meal, or a glycated hemoglobin (HbA1c) of 6.5%, and the age is 20 to 69 years old, and has been taking gemigliptin 50 mg once a day for <3 months. We also selected patients with CKD 3 (eGFR 30–59 mL/min), CKD 4 (eGFR 15–29 mL/min), or CKD 5 (eGFR < 15 mL/min, including dialysis).

Eligible participants received 50 mg/day of gemigliptin, and blood samples were collected at baseline, as well as at 3- and 6-months post-administration.

The dose of gemigliptin was fixed at 50 mg/day because this is the standard dose for treatment of type 2 diabetes in Korea. Blood tests included total lymphocyte count, lymphocyte subpopulation, regulatory T cells (CD4 + CD25 + Foxp3+), Th 17 cells, Th1/Th2 cell paired *T* test, and routine tests.

T regulatory cells were defined as CD4 + CD25 high CD127 low/- FoxP3 + phenotype, and flow cytometry was used to examine the distribution of T regulatory cells.

All participants received standard treatment for both type 2 diabetes and chronic kidney disease throughout the study. No participants discontinued gemigliptin administration during the study. The institutional review board of our center approved this study (IRB number: 2019-01-017), and written informed consent was obtained from all patients before enrollment in the study. This study was conducted in accordance with the principles of the Declaration of Helsinki.

### 2.1. [Subject inclusion criteria and exclusion criteria]

1. Inclusion criteria: (1), (2), and (3) must be included and one of (4) to (6).
  1. Fasting blood sugar 126 mg/dL or more, or 2 hours postprandial blood sugar 200 mg/dL or more, or glycated hemoglobin (HbA1c) 6.5% or more.
  2. Age: 20 to 69 years old.
  3. <3 months of taking gemigliptin 50 mg once a day.
  4. Chronic kidney disease stage 3 (eGFR 30–59 mL/min).
  5. Chronic kidney disease stage 4 (eGFR 15–29 mL/min).
  6. Chronic kidney disease stage 5 (eGFR < 15 mL/min, including patients on dialysis).
2. Exclusion criteria: Excluding any of the following cases.
  1. Chronic kidney disease stage 1 (eGFR ≥ 90 mL/min).
  2. Chronic kidney disease stage 2 (eGFR 60–89 mL/min).
  3. Type 1 diabetes.
  4. Acute renal injury (when serum creatinine rises by 0.3 mg/dL or more or 50% or more from the baseline value lasts <3 months).
  5. Hepatic failure (if alanine aminotransferase is increased more than 3 times the upper limit of normal).
  6. Acute infection.
  7. Heart failure patients: NYHA functional class II-IV patients.
  8. Patients with serious hypersensitivity reactions such as anaphylaxis or angioedema to gemigliptin or other DPP-4 inhibitors.
  9. Diabetic ketoacidosis patients.
3. Criteria for Study Suspension and Dropout.
  - In case of discontinuation of medication.
  - When the subject withdraws consent to participate in clinical research.
  - In case of transfer to another hospital.
  - Inappropriate registration: Violation of subject selection/exclusion criteria.
  - When a serious adverse reaction occurs in the subject or when the subject requests discontinuation of the test due to an adverse reaction.
  - In case the subject follow-up is unsuccessful.
  - If it is judged that the progress of the test is not suitable by the judgment of other investigators.

## 3. Flow cytometry

Isolate Peripheral Blood Monocytes (PBMCs). Dilute the blood drawn from the patient by mixing it with sterile PBS. After additional mixing of the same Ficol solution and centrifugation, the supernatant is separated and washed with PBS, and centrifugation, separation of the supernatant and washing with PBS are repeated. Check the number of PBMC, mix with dimethyl sulfoxide 10% solution, store in a refrigerator at minus 80 degrees for 24 to 72 hours, and then store in a liquid nitrogen (−150 degrees) tank.

Regulatory T cells are generated from peripheral blood mononuclear cells. Thaw and wash the frozen PBMCs, count the cells, separate the cells into one responder and one stimulator, and place them in a tube. The stimulated cell group is irradiated with 10,000 to 15,000 rad for about 2.8 minutes, mixed with 50 uL of donor peptide, and incubated for 1 hour and 30 minutes in a humidified carbon dioxide incubator at 37 degrees Celsius. After mixing low-dose IL-2, incubate for 5 to 7 days, and then perform phenotyping and functional assays through flow cytometry.

Check the distribution of T regulatory cells using the CD4 + CD25 + Regulatory T Cell Isolation Kit.

## 4. Statistical analysis

All hypotheses are tested at a significance level of 5% and, in principle, a 2-sided test is performed. All continuous variables are expressed as Mean ± standard deviation (SD) (or Median[IQR], and all nominal variables are expressed as n(%)).

A paired *T* test is performed to confirm changes in the composition of immune cells, including regulatory T cells, in type 2 diabetes patients with chronic kidney disease stage 3, 4, and 5 after taking gemigliptin 50 mg once a day at 3 months and 6 months.

The data are presented frequency with percentage for categorical variables and mean ± SD for continuous variables. Differences in study participants’ characteristics were compared across subgroups with chi-square test or Fisher exact test for categorical variables and independent test or Mann–Whitney *U* test for continuous variables as appropriate. To check if its distribution is normal, we used Shapiro–Wilk’s test.

Considering the unbalanced nature of the repeated measured data, a linear mixed model (LMM) with random intercepts was used to fit a model. The LMM model included repeated measures of numeric variables as dependent variables; group, time, and group × time interaction as fixed effects; baseline outcome as a continuous covariate; and subject as a random effect. To avoid making any assumptions about the covariance structure, we used an unstructured covariance matrix that was allowed to differ across groups for the LMM analysis, and the Bonferroni procedure was applied in post hoc analyses.

A generalized estimating equation model was used to examine differences from baseline in binary outcome between groups. The response variable is a binary variable, i.e., proportion of proteinuria (1 if positive and 0 negative); the fixed effects were time, group, and time × group interaction, and the study participants were treated as random effects. Within-subject correlation in outcomes was accounted for in the model using the appropriate covariance structure as determined by the quasi-information criteria. For graphical visualization, error bar chart and histogram were displayed.

All statistical analyses were carried out using SPSS 26.0 (IBM Corp. Released 2019. IBM SPSS Statistics for Windows, Version 26.0. Armonk, NY: IBM Corp) and *P* values <.05 was considered statistically significant.

For statistical analysis, SPSS (SPSS Inc., Chicago, IL, USA) is used, and it is considered statistically significant when *P* < .05.

## 5. Results

Of the 28 patients, 17 (60.7%) were male, with a mean age of 61.82 ± 8.03 years. Cr ≥ 1.5 mg/dL in 16 (57%) and Cr < 1.5 mg/dL in 12 (43%) (Table 1).

**Table 1:** Patient characteristics and laboratory data in diabetic patients with CKD who treatment with gemigliptin at Base line, after 3 months, 6 months.

Variable	Overall (n = 28)	Group			Analysis for repeated measures	
		CR ≥ 1.5 mg/dL (n = 16)	CR < 1.5 mg/dL (n = 12)	*P*	Source	*P*
Age (yr)						
Mean ± SD	61.82 ± 8.03	63.69 ± 5.42	59.33 ± 10.32	.2352		
Median (IQR)	63.00 (57.50–67.75)	64.00 (59.00–69.00)	62.00 (55.25–66.50)			
Gender						
Male	17 (60.7)	7 (58.3)	10 (62.5)	1.0004		
Female	11 (39.3)	5 (41.7)	6 (37.5)			
Glucose (mg/dL)						
At baseline	177.32 ± 67.91	194.75 ± 75.27a*	154.08 ± 50.63	.164‡	Group	.461
At 3 mo	173.92 ± 65.07	182.24 ± 68.47ab	156.25 ± 57.27	.268‡	Time	.463
At 6 mo	146.90 ± 46.86	135.62 ± 41.45b	167.86 ± 52.23	.147†	Group × Time	.171
BUN (mg/dL)						
At baseline	30.90 ± 13.77	36.63 ± 14.91	23.26 ± 7.11a*	.002‡	Group	.014
At 3 mo	31.96 ± 13.67	35.61 ± 14.72	24.20 ± 6.68ab	.049†	Time	.574
At 6 mo	35.92 ± 21.26	41.55 ± 24.26	25.46 ± 7.58b	.166‡	Group × Time	.895
Creatinine (CR) (mg/dL)						
At baseline	1.81 ± 0.86	2.31 ± 0.80	1.13 ± 0.26	.000†	Group	.000
At 3 mo	2.01 ± 1.03	2.46 ± 0.96	1.06 ± 0.12	.000‡	Time	.835
At 6 mo	2.09 ± 1.42	2.59 ± 1.54	1.15 ± 0.23	.000‡	Group × Time	.898
Cystatin C (mg/L)						
At baseline	1.91 ± 0.56	2.14 ± 0.60	1.61 ± 0.35	.020†	Group	.002
At 3 mo	1.96 ± 0.62	2.17 ± 0.65	1.56 ± 0.30	.032†	Time	.755
At 6 mo	2.10 ± 0.89	2.39 ± 0.98	1.55 ± 0.30	.026‡	Group × Time	.883
WBC Count (x10³/µ*l*)						
At baseline	6.89 ± 1.86	6.86 ± 1.29	6.93 ± 2.49	.9241	Group	.760
At 3 mo	7.06 ± 1.50	7.13 ± 1.10	6.92 ± 2.21	.7991	Time	.882
At 6 mo	6.80 ± 1.57	6.89 ± 1.39	6.64 ± 1.98	.7431	Group × Time	.944

* Values are the mean ± SD and Bonferroni’s post-hoc test was used for multiple comparisons between each the 3 time points. Means with different scripts are different from each other (*P* < .05).

† *P* values were derived from independent *t* test.

‡ *P* values were derived from Mann–Whitney *U* test.

Reduction in glucose in creatinine ≥ 1.5 mg/dL group was significant at 6 months compared to baseline (*P* = .020).

BUN was significantly different between groups according to creatinine (*P* = .014). It was different between groups from baseline to 3 months (*P* = .002 at baseline, *P* = .049 at 3 months) although there was no difference in BUN at 6 months (*P* = .166). Although the interaction between group and time was not significant(*P* = .895), post hoc analyses to test time effect within each group revealed the significant increase at 6 months compared to baseline in creatinine < 1.5 mg/dL group.

Cystatin C was significantly different between groups according to creatinine (*P* = .002). Cystatin C was significantly different between groups from baseline to 6 months (*P* = .020 at baseline, *P* = .032 at 3 months, *P* = .026 at 6 months). Since the interaction between group and time was not significant(*P* = .883), the 2 groups showed similar pattern in change in Cystatin C across time points.

**Figure 1.**
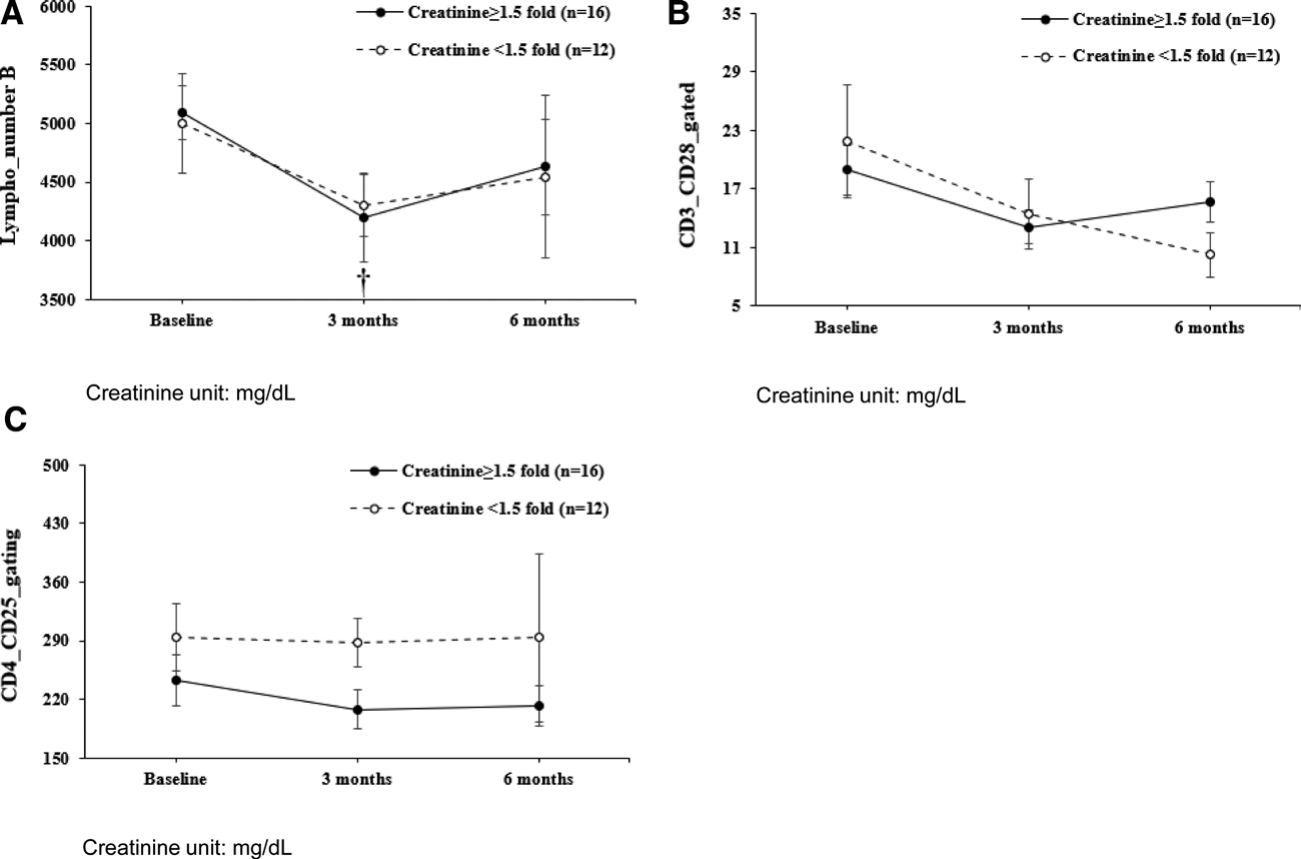
*P* < .05 Line graph. (A) Lymphocyte count, (B) CD3(+)/CD28(+) gated, (C) CD4(+)/CD25(+) gating.

Lymphocyte count was significantly decreased at 3 months compared to the overall baseline (*P* = .018) and creatinine ≤ 1.5 mg/dL group (*P* = .046) (Fig. 1A).

CD3(+)/CD28(+) gated decreased at 3 months (*P* = .039) compared to baseline (*P* = .039) (Fig. 1B). There was no significant change at 6 months (Table 2).

**Table 2:** T regulator cell CD4(+), CD3(+)/CD28(+) in diabetic patients with CKD who treatment with gemigliptin at Base line, after 3 months, 6 months.

Variable	Overall (n = 28)	Group			Analysis for repeated measures	
		CR ≥ 1.5 mg/dL (n = 16)	CR < 1.5 mg/dL (n = 12)	*P*	Source	*P*
Lympho number						
At baseline	5053.52 ± 1054.38a*	5094.38 ± 823.12a*	5000.40 ± 1344.84	.7102	Group	.849
At 3 mo	4229.68 ± 1312.24b	4193.47 ± 1525.43b	4306.63 ± 758.47	.8071	Time	.104
At 6 mo	4600.55 ± 1552.35ab	4630.62 ± 1462.37ab	4544.71 ± 1829.85	.9101	Group × Time	.894
CD4(+) gating						
At baseline	1936.17 ± 806.26	1946.46 ± 562.22	1922.80 ± 1080.38	.9511	Group	.499
At 3 mo	1703.92 ± 889.40	1689.71 ± 1043.30	1734.13 ± 471.86	.9101	Time	.582
At 6 mo	2075.75 ± 1228.61	2167.38 ± 1306.91	1905.57 ± 1145.44	.6062	Group × Time	.708
CD4(+) total (%)						
At baseline	19.36 ± 8.06	19.46 ± 5.62	19.23 ± 10.80	.9511	Group	.499
At 3 mo	17.04 ± 8.89	16.90 ± 10.43	17.34 ± 4.72	.9101	Time	.582
At 6 mo	20.76 ± 12.29	21.67 ± 13.07	19.06 ± 11.45	.6062	Group × Time	.708
CD4(+) gated (%)						
At baseline	37.54 ± 10.87	38.04 ± 9.15	36.90 ± 13.28	.8091	Group	.870
At 3 mo	38.54 ± 12.60	37.60 ± 13.87	40.54 ± 9.90	.5971	Time	.215
At 6 mo	42.96 ± 14.51	44.56 ± 16.37	40.01 ± 10.71	.5181	Group × Time	.392
CD3(+)/CD28(+) gating						
At baseline	451.70 ± 519.13	394.69 ± 280.66	525.80 ± 736.86	.664‡	Group	.870
At 3 mo	253.84 ± 194.49	257.82 ± 217.48	245.38 ± 146.49	.749‡	Time	.215
At 6 mo	301.25 ± 225.75	336.92 ± 213.27	235.00 ± 250.06	.166‡	Group × Time	.392
CD3(+)/CD28(+) total(%)						
At baseline	4.52 ± 5.19	3.95 ± 2.81	5.26 ± 7.37	.664‡	Group	.869
At 3 mo	2.54 ± 1.94	2.58 ± 2.17	2.45 ± 1.46	.749‡	Time	.212
At 6 mo	3.01 ± 2.26	3.37 ± 2.13	2.35 ± 2.50	.166‡	Group × Time	.389
CD3(+)/CD28(+) gated(%)						
At baseline	20.22 ± 13.60a*	18.93 ± 9.32	21.89 ± 18.18	.616†	Group	.861
At 3 mo	13.53 ± 7.91b	13.11 ± 6.96	14.44 ± 10.12	.703†	Time	.113
At 6 mo	13.78 ± 7.40ab	15.70 ± 7.52	10.22 ± 6.14	.285‡	Group × Time	.235

* Values are the mean ± SD and Bonferroni’s post-hoc test was used for multiple comparisons between each the 3 time points. Means with different scripts are different from each other (*P* < .05).

† *P* values were derived from independent *t* test.

‡ *P* values were derived from Mann–Whitney *U* test.

CD4(+)/CD25(+) gating was significantly different between the Cr ≥ 1.5 mg/dL and Cr < 1.5 mg/dL groups (*P* = .035), with a significant difference in the borders between the groups at 3 months (Fig. 1C). (*P* = .039).

CD4(+)/CD25(+) cell counts did not significantly increase or decrease in baseline, 3-month, and 6-month time changes. There was no significant change in the number of D127(-/FoxP3(+) cells (Table 3).

**Table 3:** T cell CD4(+), CD4(+)/CD25(+)CD127(-)/FoxP3(+) data in diabetic patients with CKD who treatment with gemigliptin at Base line, after 3 months, 6 months.

Variable	Overall (n = 28)	Group			Analysis for repeated measures	
		CR ≥ 1.5 mg/dL (n = 16)	CR < 1.5 mg/dL (n = 12)	*P*	Source	*P*
Lympho number						
At baseline	4792.96 ± 1249.21	4777.54 ± 1228.36	4813.00 ± 1342.38	.948*	Group	.329
At 3 mo	4385.28 ± 1516.64	4099.06 ± 1615.11	4993.50 ± 1141.16	.174*	Time	.660
At 6 mo	4656.20 ± 1336.87	4489.38 ± 1231.48	4966.00 ± 1566.59	.462*	Group × Time	.405
Lympho(%)						
At baseline	47.93 ± 12.49	47.78 ± 12.28	48.13 ± 13.42	.948*	Group	.578
At 3 mo	46.44 ± 18.63	44.80 ± 21.32	49.94 ± 11.41	.531*	Time	.774
At 6 mo	46.56 ± 13.37	44.89 ± 12.31	49.66 ± 15.67	.462*	Group × Time	.796
CD4(+) gating						
At baseline	1791.74 ± 795.03	1777.77 ± 642.95	1809.90 ± 996.62	.535†	Group	.466
At 3 mo	1672.92 ± 838.93	1571.65 ± 866.55	1888.13 ± 786.66	.390*	Time	.886
At 6 mo	1730.20 ± 892.75	1637.62 ± 788.87	1902.14 ± 1107.32	.542*	Group × Time	.717
CD4(+) total (%)						
At baseline	17.92 ± 7.95	17.78 ± 6.43	18.10 ± 9.97	.535†	Group	.641
At 3 mo	17.83 ± 9.90	17.33 ± 10.92	18.88 ± 7.87	.724*	Time	.946
At 6 mo	17.30 ± 8.93	16.38 ± 7.89	19.02 ± 11.07	.542*	Group × Time	.928
CD4(+) gated (%)						
At baseline	36.46 ± 9.30	36.65 ± 7.97	36.22 ± 11.26	.420†	Group	.726
At 3 mo	36.27 ± 9.71	36.16 ± 10.14	36.52 ± 9.37	.933*	Time	.975
At 6 mo	36.19 ± 11.86	35.18 ± 9.19	38.07 ± 16.42	.617*	Group × Time	.924
CD4(+)/CD25(+) gating						
At baseline	265.17 ± 117.03	242.92 ± 108.56	294.10 ± 126.98	.457†	Group	.035
At 3 mo	233.84 ± 99.30	208.29 ± 98.86	288.13 ± 80.73	.059*	Time	.781
At 6 mo	240.55 ± 168.89	211.85 ± 86.96	293.86 ± 264.76	.453*	Group × Time	.761
CD4(+)/CD25(+) total(%)						
At baseline	2.65 ± 1.17	2.43 ± 1.09	2.94 ± 1.27	.457†	Group	.066
At 3 mo	2.51 ± 1.24	2.33 ± 1.39	2.88 ± 0.81	.310*	Time	.958
At 6 mo	2.41 ± 1.69	2.12 ± 0.87	2.94 ± 2.65	.453*	Group × Time	.740
CD4(+)/CD25(+) gated(%)						
At baseline	15.73 ± 6.17	13.99 ± 5.74	17.99 ± 6.26	.126*	Group	.736
At 3 mo	19.23 ± 18.42	20.50 ± 22.22	16.55 ± 4.83	.600†	Time	.776
At 6 mo	15.28 ± 8.63	14.83 ± 7.37	16.11 ± 11.24	.761*	Group × Time	.565
CD127(-)/FoxP3(+) gating						
At baseline	66.35 ± 43.47	68.62 ± 43.83	63.40 ± 45.17	.783*	Group	.585
At 3 mo	56.68 ± 43.03	50.24 ± 38.05	70.38 ± 52.19	.284*	Time	.433
At 6 mo	53.10 ± 32.83	51.92 ± 34.61	55.29 ± 31.77	.834*	Group × Time	.639
CD127(-)/FoxP3(+) total(%)						
At baseline	0.66 ± 0.43	0.69 ± 0.44	0.63 ± 0.45	.783*	Group	.734
At 3 mo	0.61 ± 0.47	0.56 ± 0.45	0.70 ± 0.52	.489*	Time	.395
At 6 mo	0.53 ± 0.33	0.52 ± 0.35	0.55 ± 0.32	.834*	Group × Time	.803
CD127(-)/FoxP3(+) gated (%)						
At baseline	24.99 ± 14.53	26.59 ± 14.41	22.92 ± 15.18	.561*	Group	.356
At 3 mo	24.65 ± 14.08	24.59 ± 13.53	24.80 ± 16.15	.973*	Time	.510
At 6 mo	24.85 ± 12.07	25.67 ± 12.11	23.34 ± 12.78	.692*	Group × Time	.677

Variables are presented as the mean ± standard deviation and number (%). Shapiro–Wilk’s test was employed for test of normality assumption. –, not estimable.

* *P* values were derived from independent *t* test.

† *P* values were derived from Mann–Whitney *U* test.

‡ *P* values were derived by chi-square test.

§ *P* values were derived from Fisher exact test.

## 6. Discussion

In the Cr > 1.5 mg/dL group, glucose levels decreased from baseline at 6 months, showing an overall improvement of glycemic control. This shows the blood sugar lowering effect of gemigliptin.

Lymphocyte count was significantly decreased at 3 months compared to the overall baseline (*P* = .018) and creatinine ≤ 1.5 mg/dL group (*P* = .046), but there was no significant correlation at 6 months.

CD3(+)/CD28(+) gated decreased at 3 months compared to the baseline, but there was no significant difference at 6 months. CD4(+)/CD25(+) gating was significantly different between the Cr ≥ 1.5 mg/dL and Cr < 1.5 mg/dL groups (*P* = .035), with a significant difference in the borders between the groups at 3 months (Fig. 1C). There was no significant difference at 6 months. In other T reg cells, there was no significant difference between groups divided by Cr 1.5 criteria in CKD patients.

CD4(+)/CD25(+) cell counts did not significantly increase or decrease in baseline, 3-month, and 6-month time changes. There was no significant change in the number of D127(-/FoxP3(+) cells (Table 3).

In summary, the administration of gemigliptin to diabetic and CKD patients did not result in significant changes in lymphocytes, Treg cells, or their ratio.

In previous studies, DPP-4 inhibitors were suggested to impact T cell migration and reduce the number of circulating CD4 + T cells. However, in this study, no significant increase or decrease was observed in any of the CD4+/CD25+/FoxP3 + Treg cell subtypes.

In a 2015 study, it was found that Sitagliptin, a DPP-4 inhibitor, affected the subsets of circulating CD4 + T cells in patients with type 2 diabetes. After 12 weeks of Sitagliptin treatment, the number and percentage of circulating Th17 and Treg cells in patients with type 2 diabetes were reduced, but the Th17/Treg ratio remained unchanged. This reduction in CD4 + T cells, particularly Treg cells, by Sitagliptin might increase the risk of infection or autoimmune diseases, including autoimmune arthritis, in type 2 diabetes patients. However, it important to note that DPP-4 inhibitors do not impair ex vivo T cell-dependent immune function in healthy individuals or patients with type 2 diabetes.^[1]^

Gemigliptin was readily prescribed as a replacement for sitagliptin in T2DM and CKD patients, and a study was conducted. In comparison to this study, no significant increases or decreases were observed in the current study.

Kidney disease can result from immune-related injuries, involving both innate and adaptive immune responses. Resident immune cells in the kidney, such as dendritic cells, macrophages, resident lymphocytes, and circulating immune cells, play pivotal roles in the etiology and progression of kidney disease.

Kidney disease can lead to renal failure, necessitating dialysis and kidney transplantation, which is a standard lifesaving therapy for end-stage kidney disease. New immunosuppressive medications have improved short-term survival rates. However, many kidney grafts are lost within a decade due to chronic rejection, nephrotoxic effects of immunosuppressive drugs, and cumulative side effects, including heart disease, infection, cancer, and diabetes. Achieving kidney transplant tolerance is a significant clinical goal, and Tregs are being considered in both central and peripheral tolerance as a potential cellular therapy to promote long-term graft function and tolerance.^[8]^

Hence, it is crucial to consider the regulation of T-regulatory cells in patients with renal failure. If DPP4 inhibitors prove effective in maintaining regulatory T cell distribution, they may potentially reduce rejection and foster transplant tolerance in patients with diabetes and chronic kidney disease who are taking these inhibitors.

In contrast to previous studies, this research extended the follow-up period from 3 months to 6 months and examined the effects of a different DPP4 inhibitor, gemigliptin. However, it has certain limitations. There was no control group, the sample size was small, and other diabetes drugs were not considered. Furthermore, variations in patients’ medications, underlying diseases, and renal functions may have influenced the number and distribution of Treg.

As a prospective study conducted without a control group, it wasn’t possible to determine changes in the number and ratio of T reg cells in the group not administered with gemigliptin. However, these results suggest that gemigliptin may help in maintaining T reg cells and their ratio in treated patients. Therefore, continued research is necessary to explore the correlation between DPP4 inhibitors and Treg cells.

## 7. Conclusions

Treatment with gemigliptin for 3 and 6 months did not result in any significant increase or decrease in the number, percentage, or ratio of circulating Treg cells in type 2 diabetes and CKD patients. This suggests that gemigliptin administration may help maintain regulatory T cells or have no significant impact.

However, to confirm and expand upon these findings regarding the influence of DPP-4 inhibitors on T cells in patients with diabetes and CKD, large-scale, randomized prospective studies will be essential.

## Author contributions

**Conceptualization:** Ho Sik Shin, Ye Na Kim.

**Investigation:** Nagyeom Lee, Sujung Heo, Ye Na Kim, Yeonsoon Jung, Hark Rim.

**Methodology:** Nagyeom Lee, Ye Na Kim.

**Writing – original draft:** Yanghyeon Kim, Ho Sik Shin, Yeonsoon Jung, Ye Na Kim, Nagyeom Lee.

**Writing – review & editing:** Ye Na Kim, Ho Sik Shin.
